# Public Health Response to a Climate Emergency: A Teaching Exercise

**DOI:** 10.1016/j.focus.2025.100452

**Published:** 2025-09-30

**Authors:** Sheryl Bedno, John Russell, Katharine Beardmore, Pauline Thomas

**Affiliations:** 1 Division of Public Health, Bureau of Environmental and Occupational Health, Wisconsin Department of Health Services, Madison, Wisconsin; 2 Residency in Public Health and Preventive Medicine, Department of Medicine, Rutgers New Jersey Medical School, Newark, New Jersey

**Keywords:** Climate change, heat, flooding, disaster preparedness, tabletop exercise, residency training

## Abstract

Public health emergencies often involve weather-related issues, such as flooding, heatwaves, wildfires, storms, and vector-borne or water-borne diseases. Extreme weather is becoming more frequent and intense owing to climate change. Preventive medicine physicians and other public health professionals are uniquely qualified to serve in leadership or advisory roles during the actual emergencies or preparedness for the climate-related emergencies. Many residency programs are now integrating climate education into their curriculum, but few include simulated exercises. The authors present a climate-exacerbated scenario, discussion questions, and consideration for next steps in educating public health leaders and other collaborators.

## Highlights

• Training public health professionals about response to heat and flooding.
• Using a tabletop exercise to educate about climate-related emergencies.
• Developing a case that is realistic, educational, and challenging.

## Graphical Abstract

**Figure:**
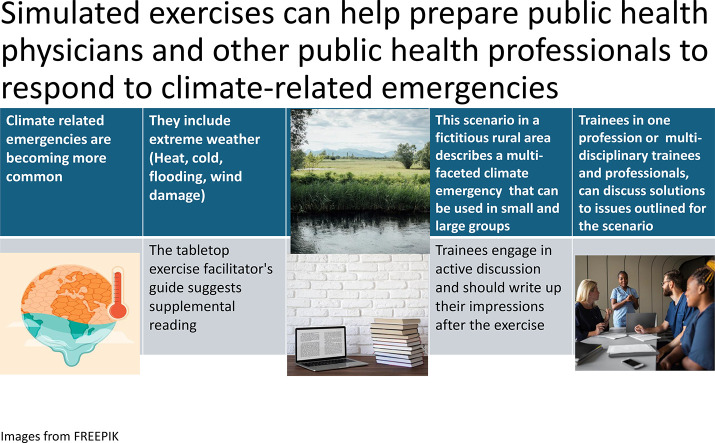

## INTRODUCTION

Climate change presents a major and escalating public health threat, intensifying hazards such as heatwaves, floods, wildfires, and vector-borne diseases, which disproportionately affect vulnerable communities and strain health systems.^1^^,^^2^ To mitigate these health impacts, robust emergency preparedness is essential, involving comprehensive planning; interagency and interdisciplinary coordination, including public health and healthcare professionals; and regular validation through practical exercises, including tabletops and drills. It is important to train the next generation of preventive medicine and other public health professionals while they are enrolled in their respective residency and training programs. In 2024, a survey of preventive medicine residency programs (occupational and environmental medicine, public health and general preventive medicine, and aerospace medicine) indicated that most taught some aspects of climate and health, but only 20% used tabletop or other exercises as part of this training.^3^

Designing impactful climate-focused public health exercises requires careful consideration of several key elements. Scenarios must be realistic and grounded in local climate projections and associated health risks and should incorporate potential cascading effects such as power outages during a heatwave or psychological distress due to displacement or material losses from flooding.^4^^,^^5^ Exercises should utilize established preparedness frameworks such as the Incident Command System and the Center for Disease Control and Prevention's Public Health Emergency Preparedness and Response Capabilities to structure objectives and guide response.^6^^,^^7^ Crucially, these exercises must include strategies to protect populations made vulnerable by SES, pre-existing conditions, or other factors, ensuring that communication and resource allocation are effective for all community members.

The ultimate aim of such preparedness exercises is to systematically evaluate and enhance the readiness of public health systems and their partners. This involves setting clear, measurable objectives and employing various exercise types, from discussion-based tabletops to operations-based drills, tailored to specific learning goals. A robust evaluation process, including participant feedback and structured After Action Reports, is vital for identifying strengths, pinpointing areas for improvement, and developing concrete corrective actions.^8^^,^^9^ By rigorously testing plans, improving coordination, and building competencies, these exercises significantly strengthen a community's ability to protect public health in the face of climate-related emergencies.^6^ The goal of this exercise is to describe a fictional but realistic case, with climate and public health implications. The exercise has been tested multiple times with medical students, preventive medicine residents, and other public health professionals. Learners acted as residents of the fictional town, with specific knowledge in preventive medicine and climate and health. Their expertise was sought by the community event planners and local government. The learning objective is to provide local decision makers with health-based recommendations and risk communication. Furthermore, the authors aim to highlight the important roles of preventive medicine physicians and other public health practitioners in public health emergencies, particularly those involving changing climate.

## CASE DEVELOPMENT

Case-based teaching can be found in many disciplines, including medicine, science, business, and others.^10^ The case method has 2 elements: the case plus the case-based discussion and analysis.^11^ The case-based methodology has many strengths, including active engagement from students or participants, improved student performance, and experience with realistic and complex situations.^12^^,^^13^

For this tabletop exercise, the aim was to develop a case and discussion questions that met the learning objectives for the audience (preventive medicine residents primarily but also medical students and public health students). The case follows the conceptual framework developed by Kim et al.^10^ with a focus on 5 attributes of cases: relevant, realistic, engaging, challenging, and instructional.

### Relevant

The case was a local scenario with a setting appropriate to the learners. It aligns with the learner’s education level and relies on the learner’s knowledge and access to resources.

### Realistic

The scenario describes a real-world setting, where learners may find themselves and requires them to use problem-solving skills that they are learning about in school or through their rotations. It provides information that may or may not be relevant to decision making, requiring them to determine what is relevant to the scenario at hand. Information is given over time as would happen in a real-world scenario.

### Engaging

The case provides some details about the community and people involved, creating a scenario that is engaging and realistic. It also requires the learners to consider multiple perspectives (themselves, government, tourism, at-risk populations, nursing facility, migrant workers, and others).

### Challenging

The case study is appropriately challenging for the learners with levels of ambiguity and withholding information until after decisions have been made, which they must adapt to. It is an unusual case for preventive medicine residents and other learners and pushes them to think differently about the system that they are working in.

### Instructional

The case study builds off prior discussions that the learners have had about weather, climate, and environmental health, reinforcing knowledge and building new connections. Assessment of the case study was conducted immediately, providing additional insights to the learners and discussing their decisions and consequences throughout. Feedback was also solicited on the case and scenario (from facilitators, faculty, learners), and changes were made to improve the experience of others.

## EXERCISE OVERVIEW

Versions of this exercise were done in person and virtually and as 1 large group or using smaller break-out groups. In all cases, the exercise began with learning objectives of the exercise and general expectations for any simulated exercise, including respect for differing points of view. The scenario and questions were distributed at the time of the exercise versus in advance. Approximately 90 minutes were allocated for initial review of the scenario followed by discussion of the various questions, but the exercise could be tailored to something shorter or longer. The case is not based in full or in part on something that previously occurred, and this was done for a few reasons. This is meant to be useful for providers or practitioners from geographically diverse areas. The scenario was written such that it could occur in many parts of the U.S. or possibly elsewhere, especially in rural areas. The authors encourage others to modify the scenario to suit their respective situations, especially outside the U.S. and in lower-resource settings. The authors also aimed to incorporate several climate-related topics plus risk factors in a single scenario, which will be presented next.

## SCENARIO

For the purposes of this case, the location, and events are fictitious and were created for educational purposes. The local public health and emergency management departments have requested a small team led by preventive medicine physicians and other healthcare and public health professionals to assist with the preparation and response to the public health emergency. One of the preventive medicine physicians happens to live in this town and has expertise and experience in climate and health and works with several other public health professionals regionally.

**Figure 1:**
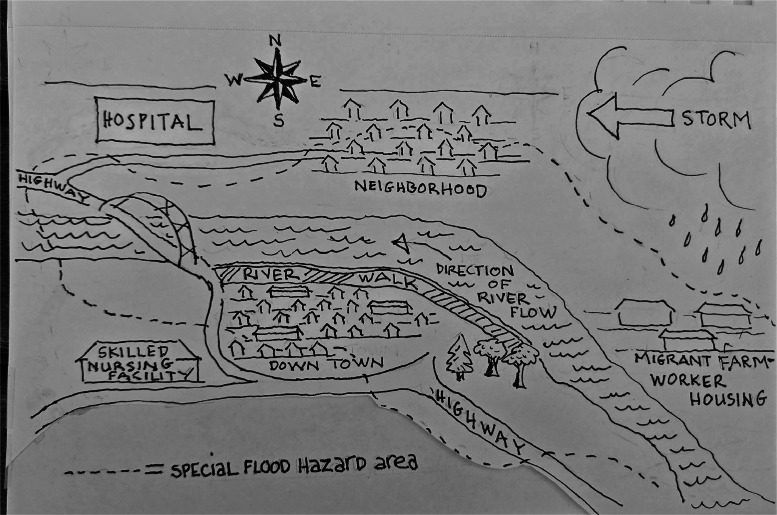
Scenario location.

The location is a small town adjacent to a river on 2 sides—to the west and to the north. The nearest hospital is to the northwest (on the other side of the river away from downtown)—it is a small hospital that serves this rural regional area (Figure 1).

The weather has been unseasonably warm in June with temperatures in the 80s. The weather service forecasts increasing temperatures (into low 90s), humidity, and high overnight temperatures from June 21 to 23. Most of the small town’s homes are older, and some do not have air conditioning because, historically, the climate has been mild in the summer. There have also been several days of rain in the first 2 weeks of June (approximately 3 inches total). The river is already elevated owing to the rain. Even more rain is forecast to start today (June 19) through June 21, with about 1–3 inches per day.

The population of the area is predominantly rural. However, there are 2 seasonal groups to note: tourists and migrant farm workers. The small town and its new riverwalk attract many tourists every summer. The town has a large event in the third week of June every year with outdoor and indoor events, commemorating local cuisine and history (the town has been planning this event all year, and it brings tourism and money to the community). This year, the event kicks off on June 22. There is a small migrant population that lives on the eastern side of the river (and they do not have air conditioning). There is 1 skilled nursing facility about 2 miles from the northwest bend in the river and across from the highway. Most of the area has a relatively high risk of flooding. The hospital is just outside this higher-risk area.

## CASE DISCUSSION

The discussion questions are divided by topic area: response preparation, first 24–48 hours of the response (immediate response), and the long-term response and impact. For some questions, there are certain answers or a set of answers expected (e.g., examples of surveillance tools), but in most cases, the questions are meant to prompt discussion among the group. Under each topic area, discussion questions and suggested responses are provided. The exercise objectives, scenario, and tables are also available in the Facilitator’s Guide (Appendix File, available online). The tables within the Facilitator’s Guide include references for further reading, for each of the questions and topic areas. Several questions are in italics because more time was spent on these during the exercises.

### Preparation

In this part of the exercise (Table 1), participants are asked whether they would recommend cancelling the large event, the annual event that this town has been preparing for over many months and that brings a lot of money and recognition to this rural community. One member of the provider team is asked to defend their position when meeting with the town’s mayor and council.

### Immediate Response

For the immediate response (Table 2), the exercise participants should presume that the town’s mayor and council decided to move forward with the annual event. The last question is an optional question related to air quality. The facilitator has the discretion to include this during this section of exercise. The topic of air quality during this scenario is realistic and adds to the complexity of the exercise.

### Long-Term Response and Impact

The longer-term impact or response (Table 3) is not often a focus of public health emergency exercises but should be incorporated given some of the longer-term consequences on health and the environment.

## EXERCISE DISCUSSION

One of the most challenging parts of the exercise is the decision whether to cancel (or modify) the town’s event and the meeting with the town’s leadership. The authors held several iterations of this exercise, and each time, the groups made different decisions (cancel, not cancel, or postpone). For the groups who decided to cancel, the authors discussed and role played how to support that decision to the town’s leadership. Given that public health recommendations or measures may not be popular or acceptable, skills in risk communication and how to speak with nonmedical audiences in the most effective ways are essential for preventive medicine physicians and others in public health leadership. The second important takeaway from the exercises is the key collaboration between preventive medicine/public health, emergency management, and others during an emergency event. Although public health is in an advisory (yet important) role, the Incident Command System and local emergency plans (e.g., heat plans) should be known. Some of the learners were not as familiar with emergency preparedness/management. Finally, there is a wealth of resources related to heat, flooding, and air quality, including surveillance tools, online resources, and others (both local and regional). Although physicians or others may not be the ones using these tools, it is important to know what is available.

## CONCLUSIONS

This scenario focuses on the detrimental impacts of heat and flooding, primarily, in a community that may have had minimal (if any) experience in aspects of climate-related emergency response in June, at the time of their annual event, or at any other time owing to the changing climate. Preventive medicine physicians are uniquely trained to advise and lead during public health emergencies, and these emergencies may be increasingly climate related or climate focused. Therefore, training related to emergency preparedness as well as climate and health must be an essential part of residency programs as well as continuing education for current physicians.^14^, ^15^, ^16^ Although this exercise was physician or resident focused, preparedness for climate-related emergencies should be collaborative and include other public health professionals, first responders, environmental health specialists, emergency managers, and those specializing in communications when possible. Effective education of preventive medicine residents, physicians, and others on climate change will involve a multitude of tools. Preparedness education using case studies and discussions such as this build essential skills for public health experts and leaders.

**Table 1:** Response Preparation.

Number	Questions	Suggested responses
1	What are the main issues to prepare for?	Heat and heat-related illnesses, flooding and conditions/diseases related to flooding, large influx in people due to the event, increased healthcare needs
2	Where would you go to find information on how to respond to these issues? Does a plan exist?	Emergency management (local, county, and/or state), plans related to heat or flooding events
3	*Do you recommend cancelling the annual event? Discuss this recommendation and how it will be received by the town’s leadership.*	Yes, we recommend cancelling, postponing, or modifying. This recommendation may not be received well by the mayor/city. Emphasis should be on health and safety of town’s residents and the visitors.
4	Name three strategies to decrease the risk of the issues stated in Question 1.	Education of providers and other health professionals, education of event planners, increased communications through different channels, availability of cooling centers/stations and shelters, identification of resilience hubs for flood displaced populations and medication storage, identification of vulnerable populations
5	What are some disease surveillance tools or other systems the team should be prepared to use?	Syndromic surveillance, contacting local providers via phone, local or state climate surveillance tools (e.g., heat vulnerability indices, flood maps)
6	With limited time, would you focus your efforts on certain vulnerable populations? If so, which ones and how?	Skilled nursing, migrants, elderly, infants and children, and those with disabilities. Public health should already have good contacts with many of these populations.

**Table 2:** Immediate Response (first 24-48 hours).

Number	Questions	Suggested responses
1	In general, what would the immediate response involve? Who would lead it?	Key part of this question is that public health/preventive medicine is not the lead but should be in an advisory role.
2	What health-related sequalae could be anticipated on June 22^nd^, the first day of the event?	Stress and anxiety, heat-related illness, physical injuries, gastrointestinal illness
3	Would there be an immediate (versus later) increase in healthcare access and utilization and what would be some of the barriers to accessing care?	Yes, there could be an immediate increase such as worried well, tourists and locals with health issues; barriers would be from flooding
4	Name three tools and resources that could be useful in the immediate response (public health or healthcare focus). Describe the tool/resource, how it is accessed, and how it will help the immediate issue(s).	Depending on circumstances, it may be a simple spreadsheet and use of a local network of providers to track healthcare utilization and types of illnesses. One could get data from emergency departments including syndromic surveillance or from emergency medical services. Given that this is a small community, ED/EMS can send aggregate data.
5	*How would you/your team balance the immediate health needs of the tourists and migrants versus the local population given limited resources?*	It may be difficult to access certain populations if roads are inaccessible. Healthcare resources will need to be prioritized based on what is life-saving/severity. Calls will need to be triaged accordingly.
6	*At around 36 hours, you are told about an increase in calls related to nausea and GI issues. How will you investigate this further? Will surveillance systems or tools be useful? If so, which ones?*	One needs to get more information. How many? Who is affected? Tourists? Migrant Farm Workers? Where do they live? Where do they spend the day? When did this start? This may or may not be related to the event(s).
7	(Added to scenario at facilitator’s discretion) Air quality is worsening in the area with an AQI nearing 100. Does this affect your planning?	Yes, if the air quality is worsening then some people may experience health effects. This will affect some at-risk populations. There will need to be messaging related to this plus some additional monitoring of health effects.

ED, emergency department; EMS, emergency medical services.

**Table 3:** Long-Term Response and Impact.

Number	Questions	Suggested responses
1	Describe the potential long-term health-related sequalae from the scenario above. List at least 3 health-related issues, the impact (individual health or community/public health), and how the issue will be resolved	Mental health (anxiety, stress) concerns: work with public health and local mental health providers; Decreased capacity to skilled nursing facility: work with neighboring facility and public health; Overall decreased healthcare access (some clinics not yet reopened): work with neighboring towns and via public health
2	What is the long-term community impact of the climate-related issues?	Events like the one experienced will become more frequent. Establishing resilience hubs to prepare for future climate-related events would be an appropriate response, leading to community resilience and acceptance of climate related changes.
3	Your team is asked to provide a report on the impact to the healthcare system, including the diseases and conditions due to the events. How would you go about this? Where would you collect data and what would you focus on?	There will be several sources of data: data from the local providers (outpatient), hospital, emergency dept/EMS, reportable disease data from local public health, syndromic surveillance, vital statistics, chamber of commerce (for tourist numbers). Important to highlight increase in population (which affects the denominator).
4	How will you determine if there are changes your team should make for future climate-related events or other emergencies? Does the mayor plan to have another annual event after this?	Ensure an after action report is done by the team, detailing what went well and didn’t go well. May wish to suggest changes to heat or flooding plan (presuming they existed in some form), or exercises before the next annual event.

EMS, emergency medical services.

## Appendix Supplementary materials

Image, application 1

## Footnotes

Supplementary material associated with this article can be found in the online version at doi:10.1016/j.focus.2025.100452.
