# Supplementary material for: Public Health Response to a Climate Emergency: A Teaching Exercise
Source: AJPM Focus. 2025 Sep 30;5(2):100452. doi: 10.1016/j.focus.2025.100452 (PMC12876716; doi:10.1016/j.focus.2025.100452)
Supplement: Supplementary file 1 [file mmc1.docx]

**Facilitator’s Guide: Public Health Response to a Climate Emergency: A Teaching Exercise**

**Learning Objectives:**

1. Compare the primary tasks in preparing versus responding to a climate-related public health emergency.
2. Discuss approaches to talking to others about the effects of climate, including those with non-scientific backgrounds and those who may disagree about the effects of climate.
3. Describe at least two tools that can be utilized during the scenario (climate-related public health emergency).
4. Identify at least three at-risk populations during the exercise scenario.

**General Expectations:**

- Be respectful and professional.
- Even if you do not agree with others, do not interrupt or degrade their contributions.
- Allow the facilitator to do their job and allow space for others to share/discuss.

**Scenario**

For the purposes of this case, the location and events are fictitious and were created for educational purposes. The local public health and emergency management departments have requested a small team led by preventive medicine physicians and other healthcare and public health professionals to assist with the preparation and response to the public health emergency. One of the preventive medicine physicians happens to live in this town and has expertise and experience in climate and health and works with several other public health professionals regionally.

The location is a small town adjacent to a river on two sides – to the west and to the north. The nearest hospital is to the northwest (on the other side of the river away from downtown) – it is a small hospital that serves this rural regional area. See Figure 1.

The weather has been unseasonably warm in June with temperatures in the 80s. The weather service forecasts increasing daytime temperatures (into low 90s), humidity, and high overnight temperatures (mid-70s) from June 21 to 23. Most of the small town’s homes are older and some do not have air conditioning, as historically the climate has been mild in the summer. There have also been several days of rain in the first two weeks of June (approximately 3 inches total). The river is already elevated due to the rain. Even more rain is forecast starting today (June 19) through June 21, with about 1-3 inches per day.

The population of the area is predominantly rural. However, there are two seasonal groups to note: tourists and farm migrant workers. The small town and its new riverwalk attract many tourists every summer. The town has a large event in the third week of June every year with outdoor and indoor events commemorating local cuisine and history (the town has been planning this event all year and it brings tourism and money to the community). This year, the event kicks off on June 22. There is a small migrant population that lives on the eastern side of the river (and they do not have air conditioning). There is one skilled nursing facility about two miles from the northwest bend in the river and across from the highway. Most of the area has a relatively high risk of flooding. The hospital is just outside this higher risk area.

**Diagram for the exercise**: this is included in the article and not repeated here. If you plan to modify the exercise scenario, please consider creating a diagram as part of your planning.

**Questions and Suggested Responses:** For each of the three topics, several questions and suggested responses are provided. The facilitator may opt to skip some of the questions depending on the direction of the discussion (i.e., the discussion is already covering some of the following questions), the allotted time for the exercise, or the focus of the exercise (the facilitator may wish to highlight certain questions more than others). Please note that these are suggested responses only. Some facilitators and groups may wish to modify the responses or even the questions depending on local circumstances (e.g., different geography or vulnerable populations, etc.). The questions in italics can take more discussion time, especially the one under Topic 1.

**Tabletop Roles**: The main roles are the participants (health professionals), overall facilitator, optional breakout group facilitator(s), and preferably notetaker (and if there are breakout groups, we recommend that someone be appointed as a notetaker for each group).

**Virtual versus In-Person**: This exercise can be conducted virtually or in-person. And with either format, it can be done in one group or in smaller groups. If the group is large (over 10 people), an option is to use break-out groups and have each group go through the questions and then have each group present their responses to the group. This option is a good way to have students or residents serve as facilitators for their small groups.

**Topic 1: Preparation**

| **#** | **Questions** | **Suggested Responses** | **For Further Reading** |
| --- | --- | --- | --- |
| 1 | What are the main issues to prepare for? | Heat and heat-related illnesses, flooding and conditions/diseases related to flooding, large influx in people due to the event, increased healthcare needs | Centers for Disease Control and Prevention (CDC). Responding to Natural Disasters and Extreme Weather.  The Medical Society Consortium on Climate and Health. Climate Resilience for Health Care Toolkit – January 2025. |
| 2 | Where would you go to find information on how to respond to these issues? Does a plan exist? | Emergency management (local, county and/or state), plans related to heat or flooding events | CDC. Emergency Preparedness and Response.  Federal Emergency Management Agency (FEMA). National Preparedness.  World Health Organization (WHO). Health Emergency and Disaster Risk Management Framework. |
| 3 | *Do you recommend cancelling the annual event? Discuss this recommendation and how it will be received by the town’s leadership.* | Yes, we recommend cancelling, postponing, or modifying. This recommendation may not be received well by the mayor/city. Emphasis should be on health and safety of town’s residents and the visitors. | DeBree et al 2024.  Global Heat Health Information Network. Communicate & Advocate. |
| 4 | Name three strategies to decrease the risk of the issues stated in Question 1. | Education of providers and other health professionals, education of event planners, increased communications through different channels, availability of cooling centers/stations and shelters, identification of resilience hubs for flood displaced populations and medication storage, identification of vulnerable populations | CDC. Climate and Health Strategic Framework.  US Environmental Protection Agency (EPA). Public Health Resiliency Strategies. |
| 5 | What are some disease surveillance tools or other systems the team should be prepared to use? | Syndromic surveillance, contacting local providers via phone, local or state climate surveillance tools (e.g., heat vulnerability indices, flood maps) | CDC. Public Health Surveillance During a Disaster.  World Health Organization. Climate and Health. |
| 6 | With limited time, would you focus your efforts on certain vulnerable populations? If so, which ones and how? | Skilled nursing, migrants, elderly, infants and children, and those with disabilities. Public health should already have good contacts with many of these populations. | CDC. Planning for an Emergency: Strategies for Identifying and Engaging At-Risk Group.  EPA. Climate Change and the Health of Socially Vulnerable People. |

**Topic 2: Immediate Response (first 24-48 hours)** (Presume that town’s event was not cancelled)

| **#** | **Questions** | **Suggested Responses** | **For Further Reading** |
| --- | --- | --- | --- |
| 1 | In general, what would the immediate response involve? Who would lead it? | Key part of this question is that public health/preventive medicine is not the lead but should be in an advisory role. | NACCHO. Local health departments prepare for and respond to emergencies.  Clark-Ginsberg at al 2024. |
| 2 | What health-related sequalae could be anticipated on June 22^nd^, the first day of the event? | Stress and anxiety, heat-related illness, physical injuries, gastrointestinal illness | CDC. Floods.  Mental Health America. Coping with Disaster. |
| 3 | Would there be an immediate (versus later) increase in healthcare access and utilization and what would be some of the barriers to accessing care? | Yes, there could be an immediate increase such as worried well, tourists and locals with health issues; barriers would be from flooding | Wang et al 2023. |
| 4 | Name three tools and resources that could be useful in the immediate response (public health or healthcare focus). Describe the tool/resource, how it is accessed, and how it will help the immediate issue(s). | Depending on circumstances, it may be a simple spreadsheet and use of a local network of providers to track healthcare utilization and types of illnesses. One could get data from emergency departments including syndromic surveillance or from emergency medical services. Given that this is a small community, ED/EMS can send aggregate data. | CDC. Public Health Surveillance During a Disaster.  Foege. House on Fire: The Fight to eliminate smallpox. |
| 5 | *How would you/your team balance the immediate health needs of the tourists and migrants versus the local population given limited resources?* | It may be difficult to access certain populations if roads are inaccessible. Healthcare resources will need to be prioritized based on what is life-saving/severity. Calls will need to be triaged accordingly. | Bazyar et al 2019.  Lee 2010. |
| 6 | *At around 36 hours, you are told about an increase in calls related to nausea and GI issues. How will you investigate this further? Will surveillance systems or tools be useful? If so, which ones?* | One needs to get more information. How many? Who is affected? Tourists? Migrant Farm Workers? Where do they live? Where do they spend the day? When did this start? This may or may not be related to the event(s). | CDC. Public Health Surveillance During a Disaster. |
| 7 | (Added to scenario at facilitator’s discretion) Air quality is worsening in the area with an AQI nearing 100. Does this affect your planning? | Yes, if the air quality is worsening then some people may experience health effects. This will affect some at-risk populations. There will need to be messaging related to this plus some additional monitoring of health effects. | CDC. Air Quality. Resources for Professionals. |

**Topic 3: Long-Term Response and Impact**

| **#** | **Questions** | **Suggested Responses** | **For Further Reading** |
| --- | --- | --- | --- |
| 1 | Describe the potential long-term health-related sequalae from the scenario above. List at least 3 health-related issues, the impact (individual health or community/public health), and how the issue will be resolved | Mental health (anxiety, stress) concerns: work with public health and local mental health providers;  Decreased capacity to skilled nursing facility: work with neighboring facility and public health;  Overall decreased healthcare access (some clinics not yet reopened): work with neighboring towns and via public health | Heanoy et al 2024.  Gu et al 2025.  Keenan et al 2025.  Saito et al 2025.  Valavani et al 2025.  Sands et al 2022. |
| 2 | What is the long-term community impact of the climate-related issues? | Events like the one experienced will become more frequent. Establishing resilience hubs to prepare for future climate-related events would be an appropriate response, leading to community resilience and acceptance of climate related changes. | Fundter et al 2012 (TAP report). |
| 3 | Your team is asked to provide a report on the impact to the healthcare system, including the diseases and conditions due to the events. How would you go about this? Where would you collect data and what would you focus on? | There will be several sources of data: data from the local providers (outpatient), hospital, emergency dept/EMS, reportable disease data from local public health, syndromic surveillance, vital statistics, chamber of commerce (for tourist numbers). Important to highlight increase in population (which affects the denominator). | Miri et al 2024. |
| 4 | How will you determine if there are changes your team should make for future climate-related events or other emergencies? Does the mayor plan to have another annual event after this? | Ensure an after action report is done by the team, detailing what went well and didn’t go well. May wish to suggest changes to heat or flooding plan (presuming they existed in some form), or exercises before the next annual event. | WHO Health Emergency Dashboard. |

**For Further Reading (References)**

1. CDC. Responding to Natural Disasters and Extreme Weather. Emergency Preparedness and Response. July 28, 2025. Accessed September 21, 2025. https://www.cdc.gov/niosh/emres/response/index.html

2. Martincic SM. Climate Resilience for Health Care (CR4HC) Toolkit. The Medical Society Consortium on Climate and Health (MSCCH). January 7, 2025. Accessed September 21, 2025. https://medsocietiesforclimatehealth.org/advocacy-resources/climate-resilience-for-health-care-cr4hc-toolkit/

3. CDC. Emergency Preparedness and Response. Emergency Preparedness and Response. August 13, 2025. Accessed September 21, 2025. https://www.cdc.gov/emergency/index.html

4. National Preparedness | FEMA.gov. February 3, 2025. Accessed September 21, 2025. https://www.fema.gov/emergency-managers/national-preparedness

5. Health Emergency and Disaster Risk Management Framework. Accessed September 21, 2025. https://www.who.int/publications/i/item/9789241516181

6. DeBree SH, Southwell BG, Brown JA. How health risk communication best practices can improve community flood risk communication. *Journal of Public Health and Emergency*. 2024;8(0). doi:10.21037/jphe-24-27

7. Communicate and Advocate | Global Heat Health Information Network. July 13, 2020. Accessed September 21, 2025. https://heathealth.info/communicate/, https://heathealth.info/communicate/

8. CDC. Climate and Health. Climate and Health. July 2, 2024. Accessed September 21, 2025. https://www.cdc.gov/climate-health/index.html

9. US EPA O. Public Health Resiliency Strategies. April 28, 2016. Accessed September 21, 2025. https://www.epa.gov/arc-x/public-health-resiliency-strategies

10. CDC. Public Health Surveillance During a Disaster. Disaster Epidemiology and Response. September 10, 2024. Accessed September 21, 2025. https://www.cdc.gov/disaster-epidemiology-and-response/php/disaster/surveillance.html

11. Integrated surveillance and climate-informed health early warning systems. Accessed September 21, 2025. https://www.who.int/teams/environment-climate-change-and-health/climate-change-and-health/country-support/integrated-surveillance-and-climate-informed-health-early-warning-systems

12. Planning for an Emergency: Strategies for Identifying and Engaging At-Risk Groups. Accessed September 21, 2025. https://www.cdc.gov/disaster-epidemiology-and-response/media/pdfs/2025/01/atriskguidance_updated.pdf

13. US EPA O. Climate Change and the Health of Socially Vulnerable People. March 21, 2022. Accessed September 21, 2025. https://www.epa.gov/climateimpacts/climate-change-and-health-socially-vulnerable-people

14. NACCHO Local health Departments Prepare for and Respond to Emergencies. Accessed September 21, 2025. https://www.malph.org/sites/default/files/DONE%20NACCHO%20Emergency%20Preparedness%202019.pdf

15. Clark-Ginsberg A, Fisher H, Awan J, et al. Conceptual Framework for Understanding Incident Management Systems During Public Health Emergencies. *Disaster Med Public Health Prep*. 2022;17:e158. doi:10.1017/dmp.2022.77

16. CDC. Safety Guidelines: Floodwater. Floods. September 30, 2024. Accessed September 21, 2025. https://www.cdc.gov/floods/safety/floodwater-after-a-disaster-or-emergency-safety.html

17. Coping With Disaster | Mental Health America. Accessed September 21, 2025. https://mhanational.org/resources/coping-with-disaster/

18. Wang W, Li H, Huang M. A literature review on the impact of disasters on healthcare systems, the role of nursing in disaster management, and strategies for cancer care delivery in disaster-affected populations. *Front Oncol*. 2023;13:1178092. doi:10.3389/fonc.2023.1178092

19. House on Fire by William Foege - Paper. University of California Press. Accessed September 21, 2025. https://www.ucpress.edu/books/house-on-fire/paper

20. Bazyar J, Farrokhi M, Khankeh H. Triage Systems in Mass Casualty Incidents and Disasters: A Review Study with A Worldwide Approach. *Open Access Maced J Med Sci*. 2019;7(3):482-494. doi:10.3889/oamjms.2019.119

21. Lee CH. Disaster and Mass Casualty Triage. *AMA Journal of Ethics*. 2010;12(6):466-470. doi:10.1001/virtualmentor.2010.12.6.cprl1-1006

22. CDC. Resources for Professionals. Air Quality. April 21, 2024. Accessed September 21, 2025. https://www.cdc.gov/air-quality/php/resources/index.html

23. Heanoy EZ, Brown NR. Impact of Natural Disasters on Mental Health: Evidence and Implications. *Healthcare (Basel)*. 2024;12(18):1812. doi:10.3390/healthcare12181812

24. Gu KM, Lee T, Myong JP. Wildfire Exposure and Respiratory Health: A Comprehensive Review of Emerging Evidence. *Tuberc Respir Dis (Seoul)*. Published online August 26, 2025. doi:10.4046/trd.2025.0064

25. Keenan OJ, Soroka O, Abramson D, Safford M, Shapiro MF, Ghosh AK. Long-term impacts of hurricanes on mortality among Medicare beneficiaries: evidence from Hurricane Sandy. *Front Public Health*. 2025;13. doi:10.3389/fpubh.2025.1523941

26. Sands LP, Do Q, Du P, Pruchno R. Peritraumatic Stress From a Disaster Increases Risk for Onset of Chronic Diseases Among Older Adults. *Innov Aging*. 2022;6(1):igab052. doi:10.1093/geroni/igab052

27. Valavani E, Bellos V, Apostolidou-Kiouti F, Benos A, Kondilis E. All-cause excess mortality following Storm Daniel flood event in Greece. *J Public Health (Oxf)*. 2025;47(2):217-221. doi:10.1093/pubmed/fdaf015

28. Saito H, Murakami M, Ozaki A, et al. Estimating the impact of missed colorectal cancer diagnoses on life expectancy in Minamisoma City following the 2011 triple disaster. *PLoS One*. 2025;20(6):e0324822. doi:10.1371/journal.pone.0324822

29. Fundter DQP, Jonkman B, Beerman S, et al. Health Impacts of Large-Scale Floods: Governmental Decision-Making and Resilience of the Citizens. *Prehospital and Disaster Medicine*. 2008;23(S2):s70-s73. doi:10.1017/S1049023X00021282

30. Miri J, Atighechian G, Seyedin H, Raeisi AR. Components and entities of post-disaster damage and loss assessment program in healthcare sector: a scoping review. *BMC Public Health*. 2024;24(1):2417. doi:10.1186/s12889-024-19523-5

31. After Action Review | Strategic Partnership for Health Security and Emergency Preparedness (SPH) Portal. Accessed September 21, 2025. https://extranet.who.int/sph/aar

**Note on References:** These are just a few of the many references on the topics addressed in the scenario and exercise and we, the authors, encourage you to explore the many other resources on this topic.

**Modification of this Guide:** The authors encourage use and modification of the contents of this guide as needed to suit your geographical or local circumstances.

Thank you –

The authors (Sheryl Bedno, John Russell, Katharine Beardmore, Pauline Thomas)
